# Synthetic Proteins and Peptides for the Direct Interrogation of α-Synuclein Posttranslational Modifications

**DOI:** 10.3390/biom5031210

**Published:** 2015-06-25

**Authors:** Matthew R. Pratt, Tharindumala Abeywardana, Nicholas P. Marotta

**Affiliations:** 1 Department of Chemistry, University of Southern California, Los Angeles, CA 90089, USA; E-Mails: abeyward@usc.edu (T.A.); nmarotta@usc.edu (N.P.M.); 2 Department of Molecular and Computational Biology, University of Southern California, Los Angeles, CA 90089, USA

**Keywords:** Synuclein, posttranslational modifications, synthesis

## Abstract

α-Synuclein is the aggregation-prone protein associated with Parkinson’s disease (PD) and related neurodegenerative diseases. Complicating both its biological functions and toxic aggregation are a variety of posttranslational modifications. These modifications have the potential to either positively or negatively affect α-synuclein aggregation, raising the possibility that the enzymes that add or remove these modifications could be therapeutic targets in PD. Synthetic protein chemistry is uniquely positioned to generate site-specifically and homogeneously modified proteins for biochemical study. Here, we review the application of synthetic peptides and proteins towards understanding the effects of α-synuclein posttranslational modifications.

## 1. Introduction

**Figure 1:**
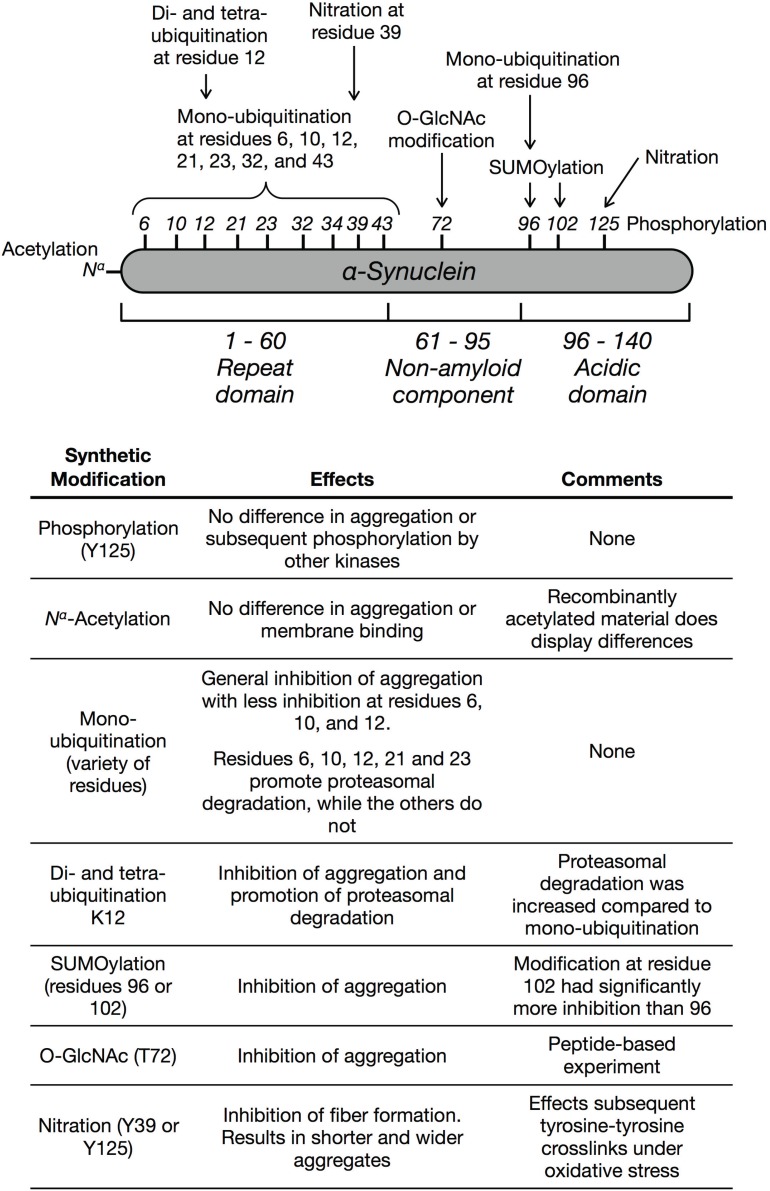
Schematic representation of the protein α-synuclein, consisting of an N-terminal repeat domain, the non-amyloid beta component domain that is required for aggregation, and a C-terminal acidic domain. The sites and identities of posttranslational modifications that have been studied using synthetic proteins or peptides are highlighted and the general effects are tabulated.

As biomedical advances lead to extensions in average life-expectancy, understanding age-related neurodegenerative diseases becomes increasingly important, particularly in the industrialized world. Alzheimer’s disease alone was the sixth leading cause of death in the United States in 2008 [1]. Unfortunately, treatments for these diseases are only palliative and effective diagnostic, preventative, and curative therapeutic options are needed [2]. Several of these diseases, termed synucleinopathies (e.g., Parkinson’s disease (PD)), are characterized by the accumulation of protein aggregates, Lewy bodies and Lewy neurites, in the brains of patients. It is estimated that ~50,000 cases of PD are diagnosed every year in the United States, with a total of at least 500,000 cases in 2006, and since the disease is not usually diagnosed until the onset of visible symptoms, this number most likely underestimates the number of people living with the disease. Given the devastating nature of the disease and the high societal and economic costs, considerable research effort has enabled the identification of several proteins that are potential therapeutic targets in PD. One of the most direct and therefore attractive targets is α-synuclein, a 140 amino acid protein that is the major component of Lewy bodies and highly expressed in pre-synaptic neurons of the central nervous system (Figure 1) [3]. Native α-synuclein exists as an unfolded monomer in the cytosol [4], and it adopts an extended α-helical conformation when associated with cellular membranes [5]. More recently, there was a report of a soluble α-helical tetramer that was purified from *E. coli* or isolated from mammalian cells [6,7]. However, a very detailed analysis by others has called these results into question, suggesting that the α-helical tetramer may result from interactions with membranes or other proteins [8]. The sequence of α-synuclein is comprised of three domains. The N-terminal region (residues 1–60) contains lysine-rich, repetitive segments that are critical for interactions with membranes. This is followed by non-amyloid beta component (NAC), compromising amino acids 61–95, which is made up of predominantly hydrophobic residues and is required for α-synuclein aggregation. Finally, residues 96–140 make up the acidic C-terminal region that is consistently found in a disordered conformation and has been implicated in interactions with proteins [9,10,11], metals [12], and small molecules [13]. A pathological role for α-synuclein in PD was first demonstrated using human genetic screening. Several point mutations (e.g., A30P, A53T, and E46K) and multiplications of the gene encoding α-synuclein (*SNCA*) result in early onset, familial PD [14,15,16,17,18]. In diseased cells, α-synuclein consists of β-sheet rich, high molecular-weight aggregates [19]. Notably, this disease state closely resembles the oligomers and fibrils that are readily formed by α-synuclein *in vitro*. Additionally, a range of oligomers, fibrils, and aggregates formed *in vitro* are highly toxic to cells and model organisms, biochemically supporting their role in disease [20,21,22]. Finally, α-synuclein can be transferred intercellularly via endocytosis where it can propagate disease pathology [23,24,25]. For example, when fibers of α-synuclein formed *in vitro* were injected into the brains of healthy, wild-type mice, intracellular aggregates were formed, and these aggregates spread throughout the brain over time [26]. Notably, these mice also developed motor defects that correspond to PD symptoms in humans.

Taken together, these data strongly support the pursuit of inhibitors of α-synuclein aggregation as promising PD therapeutics. However, a complete understanding of this process *in vivo* is complicated by a variety of α-synuclein posttranslational modifications including proteolysis [27], phosphorylation [28,29,30,31,32], ubiquitination [33], glycosylation [34,35,36], and others [37]. Many of these modifications have been identified *in vivo* and in Lewy bodies isolated from patients, and therefore understanding their effects on α-synuclein aggregation and toxicity is essential for a complete picture of PD. Furthermore, if specific posttranslational modifications can be identified that either promote or inhibit α-synuclein aggregation, the enzymes that are responsible for their respective installation or removal would be potential targets for PD drug development. Here, we review how peptides and synthetic proteins have contributed to the understanding of α-synuclein posttranslational modifications. For a more general review of α-synuclein posttranslational modifications and biochemical and genetic experiments aimed at understanding their functions, we direct to reader to other excellent reviews [3,37,38].

## 2. Protein (Semi)Synthesis by Native and Expressed Protein Ligation

**Figure 2:**
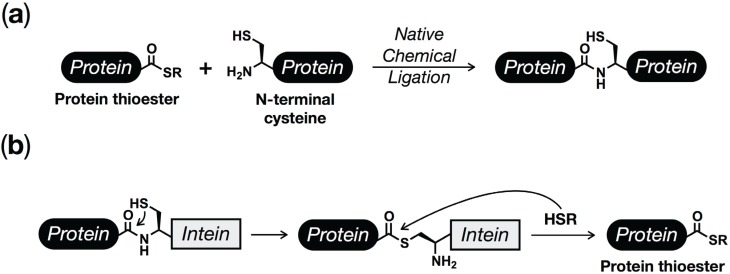
Protein synthesis using ligation chemistries. (**a**) Native Chemical Ligation (NCL) enables the selective reaction of protein or peptide thioesters with N-terminal cysteine residues, allowing for the generation of native peptides bonds under physiological conditions and without any protecting groups. (**b**) Inteins can be exploited to generate recombinant protein thioesters that can be used in NCL reactions, which has been termed Expressed Protein Ligation (EPL).

Chemical synthesis has long played a key role in the discovery of biological processes through the production of homogeneous, structurally-defined materials. A major step towards extending the power of chemical synthesis to proteins came from the development of native chemical ligation (NCL, Figure 2a) [39]. Using this method, two unprotected peptides can be reacted with each other under neutral aqueous conditions, resulting in the formation of a native peptide bond. The first step of NCL involves the reversible reaction between a C-terminal thioester on one peptide and an N-terminal cysteine residue on a second peptide. Once formed this inter-peptide thioester bond collapses by a spontaneous S to N acyl shift, resulting in an amide bond. Because the first step of this process is reversible, NCL is compatible with the side-chain functionalities of all the naturally occurring amino acids including cysteine. Additionally, proteins can be prepared semi-synthetically using NCL by reacting synthetic peptide-thioesters with recombinantly expressed proteins containing N-terminal cysteines. While large proteins have been generated using only NCL, the technique is inherently constrained in the preparation of peptide-thioesters by the limitations of solid-phase peptide synthesis (SPPS).

A solution to this problem required the preparation of recombinant protein-thioesters, which was realized by exploiting a naturally occurring process known as protein splicing. Protein splicing is a natural, posttranslational process where an internal protein segment, termed an intein, is removed and the two flanking regions, referred to as exteins, are ligated together with an amide bond [40,41,42]. The first step of protein splicing with a typical intein involves a cysteine protease-like transfer of the N-terminal extein to the side chain of a cysteine residue found at the immediate N-terminus of the intein. This is followed by a trans-thioesterification reaction where the N-terminal extein is moved to another cysteine residue at the C-terminal extein/intein junction. This branched intermediate is resolved by cyclization of a conserved asparagine residue located at the C-terminus of the intein, resulting in excision of the intein. The final step of the process is essentially identical to the second step of NCL, where a S to N acyl shift ligates the two exteins together in a native peptide bond. Although the biological role of protein splicing is still under active investigation [43], the reaction mechanism can be exploited to generate protein thioesters for NCL. Specifically, a number of intein mutants have been developed that can only undergo the first step of protein splicing [44]. Proteins recombinantly expressed as N-terminal fusions to one of these inteins can be cleaved by the addition of exogenous thiols to generate protein thioesters that can partake in NCL reactions in a process termed expressed protein ligation (EPL, Figure 2b) [45,46,47,48], which enables the systematic incorporation of synthetic peptides into large proteins.

## 3. Phosphorylation

Phosphorylation results from the transfer of the ɣ-phosphate from ATP to protein substrates by over 500 kinases and can occur on serine and threonine or tyrosine residues. The first data supporting the phosphorylation of α-synuclein came from cell culture experiments which identified S87 and S129 as potential sites of modification *in vivo* [49]. This was quickly followed by the development of phosphorylation-specific antibodies and demonstration that S129 phosphorylated α-synuclein is a major component of aggregates in patients who had succumbed to PD, multiple system atrophy, and dementia with Lewy bodies [28,50,51]. Furthermore, phosphorylation at S129 correlates consistently with aggregates in a variety of cellular and animal model studies. More recently, α-synuclein was also shown to be phosphorylated at Y125 *in vivo* [31], and S87 phosphorylation was found to be enhanced in aggregates [30]. Different genetic and biochemical experiments have yielded somewhat contradictory evidence concerning the molecular consequences of α-synuclein phosphorylation [37]. For example, animal model studies have reached different conclusions about S129 phosphorylation depending on whether the study utilized a mutant (S129A or S129D/E) of α-synuclein or overexpression of a kinase known to phosphorylate the protein at S129 [52,53,54]. For example, *in vitro* phosphorylation of α-synuclein using casein kinase II (CK2) behaved differently than either S129A or S129E/D mutants [29]. More recently, a model has emerged that could explain these results [29,54,55]. The Lashuel lab demonstrated that phosphorylation at S129 and S87 or S129 alone inhibits α-synuclein aggregation and that certain kinases can also phosphorylate pre-formed aggregates at S129, explaining why it is closely associated with aggregates in model systems. Additionally, they found that S129 phosphorylation promotes the degradation of α-synuclein. This highlights the importance of homogeneously phosphorylated material for understanding the fundamental consequences of this modification.

To date, only one phosphorylation site, Y125, has been explored using synthetic proteins. Using protein semisynthesis, the Lashuel lab prepared α-synuclein phosphorylated at Y125 from a recombinant thioester (residues 1–106) and a synthetic peptide (residues 107–140). Comparing this phosphorylated protein to the unmodified, recombinant control demonstrated that both proteins had similar binding to membranes (CD spectroscopy) and the same overall structure by NMR. Notably, the authors also found no difference in the aggregation of unmodified and phosphorylated α-synuclein by thioflavin T fluorescence, SDS-PAGE analysis, or transmission electron microscopy. After microinjection into neurons, the authors also observed rapid dephosphorylation of the protein within 30 min. Finally, they found that phosphorylation at Y125 had little to no effect on the subsequent phosphorylation of α-synuclein by polo-like kinase 3 (PLK3) at S87 or S129. Although this phosphorylation site displayed little difference in a handful of experiments, it does highlight the biochemical discoveries that can be obtained using synthetic proteins.

## 4. Acetylation

The majority of proteins in mammalian cells are modified at their *N*-termini by acetylation (*N*^α^-acetylation) [56,57]. *N*^α^-Acetylation is installed by a family of acetyltransferases that use acetyl-CoA as a substrate [58]. Analysis of both soluble α-synuclein and Lewy bodies has demonstrated that the protein is constitutively *N*^α^-acetylated [51]; however, the majority of *in vitro* experiments are performed with recombinant α-synuclein that does not bear this modification. To determine if *N*^α^-acetylation affects the biophysical properties of α-synuclein, the Lashuel lab used expressed protein ligation to prepare *N*^α^-acetylated protein [59]. Using a variety of assays to measure the structure, aggregation, and membrane interactions of this protein, they were able to determine that *N*^α^-acetylation has little to no effect on α-synuclein, indicating that recombinant protein could be appropriate for most α-synuclein studies. Notably, however, several other studies have used *in situ* acetylation in recombinant expression systems and have found that *N*^α^-acetylation can have effects on the induction of α-helicity in the N-terminus, interactions between α-synuclein and membranes, and oligomer and aggregate formation [60,61,62,63]. Therefore, more studies are needed to understand the role of this modification *in vivo*, both in terms of the endogenous functions of α-synuclein and in PD.

## 5. Ubiquitination

The vast majority of ubiquitin modifications result from the enzymatic addition of the small protein (76 residues) ubiquitin, through its C-terminus, to the side chain amine of substrate protein lysine residues, giving an isopeptide bond [64]. The enzymatic addition of ubiquitin to substrate proteins usually involves three different steps. First, ubiquitin is activated as a C-terminal thioester through conjugation to an E1 ubiquitin activating enzyme [65]. The ubiquitin can then be transferred to an E2 ubiquitin conjugating enzyme [66]. The E2 can then be brought into close proximity with a substrate protein by a RING domain E3 ubiquitin ligase, resulting in ubiquitin transfer [67]. Alternatively, HECT domain E3 ubiquitin ligases can first accept ubiquitin form an E2 enzyme on their own catalytic cysteine residue before transferring it to a substrate protein [68]. This first ubiquitin modification (mono-ubiquitination) can then be polymerized to form poly-ubiquitin chains. This polymerization can occur on seven different lysine residues and the N-terminus of the acceptor ubiquitin, resulting in an array of different molecular structures with potentially unique functions [69]. Ubiquitination is also dynamic, as there are a range of proteases (deubiquitinases) that can remove the protein from its substrates [70,71].

A potential role for ubiquitination of α-synuclein in PD was first uncovered through a variety of pathology studies that demonstrated ubiquitin-positive staining in Lewy bodies [33,72,73,74,75,76,77]. Biochemical analysis demonstrated that α-synuclein is directly modified by ubiquitin in Lewy bodies and that the vast majority of the modification is mono-ubiquitination, with some *di*- and *tri*-ubiquitination [51,75,76,78]. A combination of *in vitro* and *in vivo* experiments showed that α-synuclein can be mono-ubiquitinated at 9 out of 15 lysine residues. Ubiquitination of monomeric, recombinant α-synuclein using fraction II of rabbit reticulocytes resulted in mono-ubiquitination of K21, K23, K32, and K34 [78]. Notably, the same experiment with pre-formed α-synuclein fibers yielded modification of K6, K10, and K12 [78]. Subsequent discovery of the major E3 ubiquitin ligases responsible for α-synuclein mono-ubiquitination, seven of absentia homolog 1/2 (SIAH1/2), enabled *in vitro* biochemical and cell culture experiments [79,80]. These studies confirmed previous results and showed that these ligases can mono-ubiquitinate α-synuclein at K6, K10, K12, K21, K23, K32, K34, K46, and K96. Using traditional biological techniques has produced somewhat contradictory evidence for the consequences of α-synuclein mono-ubiquitination on aggregation and toxicity. Overexpression of SIAH1/2 in PC12 and SH-SY5Y cells increases the formation of α-synuclein aggregates [79,80], and *in vitro* ubiquitination by SIAH promotes the formation of large aggregates as visualized by electron microscopy [79]. However, animal models of PD that genetically upregulate ubiquitination show a protective role against α-synuclein toxicity [81]. Furthermore, *in vitro* experiments demonstrated that the E3 ubiquitin ligase NEDD4 will heterogeneously modify α-synuclein with 63-linked polyubiquitin chains, and the same study found that overexpression of NEDD4 lead to the lysosomal degradation of α-synuclein in cell culture [82].

**Figure 3:**
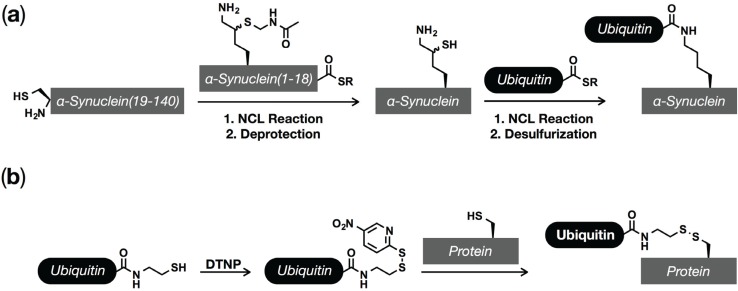
Chemical methods for the installation of ubiquitin and ubiquitin-like modifications. (**a**) Synthesis with δ-mercaptolysine. A synthetic peptide thioester containing a protected δ-mercaptolysine is reacted with a recombinant protein fragment to generate full-length α-synuclein. After deprotection of the δ-mercaptolysine residue, it can be selectively reacted with a recombinant ubiquitin thioester. Desulferization then gives site-specifically ubiquitination α-synuclein with no mutations. (**b**) Disulfide-directed ubiquitination. A C-terminal ubiquitin thiol, produced using intein chemistry, can be activated as a mixed disulfide, which can then be transferred to a cysteine residue on α-synuclein.

In an effort to determine the consequences of site-specific ubiquitination on α-synuclein aggregation, the Lashuel and Brik labs collaborated to first synthesize and then characterize α-synuclein bearing mono-ubiquitination at lysine 6 (Figure 3a) [83]. To prepare the synthetic protein, they took advantage of a lysine analog, δ-mercaptolysine, which will readily undergo NCL reactions on its side-chain after its incorporation into a peptide or protein [84]. Specifically, this amino acid was first placed at residue 6 into a thioester peptide corresponding to the first 19 amino acids of α-synuclein by solid-phase peptide synthesis. This thioester peptide then underwent an NCL reaction with an N-terminal cysteine containing recombinant protein to generate full-length α-synuclein. After a protecting group manipulation, EPL with an intein-generated ubiquitin thioester installed ubiquitin at lysine 6. Since α-synuclein has no native cysteine residues, the thiol groups required for the ligation reactions were then removed by desulfurization to yield site-specifically ubiquitinated α-synuclein with no mutations. Using this synthetic protein, the authors demonstrated that ubiquitination at lysine 6 inhibited protein aggregation but had no effect on the phosphorylation of α-synuclein at either S87 or S129, indicating that ubiquitination may have a beneficial role in PD. Although this elegant strategy produces ubiquitinated protein with no mutations or other structural changes, it is synthetically challenging, making the synthesis of the other 8 α-synuclein modification sites difficult. To overcome this issue, we took advantage of a complementary technique, termed disulfide-directed ubiquitination, that replaces the native lysine iso-peptide linkage with a disulfide analog [85,86]. Using this technique, we prepared α-synuclein with site-specific ubiquitination at each of the nine potential modification-sites [87]. In agreement with the work by Lashuel and Brik, we found that most ubiquitination sites delay aggregation; however, they are not all equally potent, with modification sites towards the center of the protein generally exhibiting a higher degree of inhibition, consistent with the previously observed enzymatic ubiquitination of lysines 6, 10, and 12 in pre-formed fibers [78].

The canonical role for ubiquitination is the targeted degradation of substrate proteins by the proteasome. This is typically mediated by lysine-48 (K48) linked poly-ubiquitin chains that are the most common form of polyubiquitin found in cells; however, recently, Ciechanover and coworkers demonstrated that short proteins (less than 150 amino-acids) only require mono-ubiquitination to mediate proteasomal turnover [88]. In this same study, α-synuclein bearing mono-ubiquitination at lysine 12 was prepared using δ-mercaptolysine in the same way as lysine 6 above, and this protein was degraded by the proteasome *in vitro*, while unmodified α-synuclein was not. More recently, the same labs synthetically prepared α-synuclein with di- and tetra-ubiquitin (lysine 48-linked chains) at lysine 12, which largely blocked the formation of fibers and promoted proteasomal degradation more efficiently than mono-ubiquitination [89]. We also explored the degradation of mono-ubiquitinated α-synuclein using the disulfide-directed ubiquitination approach (Figure 3b) [90]. As above, mono-ubiquitinated α-synuclein disulfide-analogs were prepared corresponding to each of nine modification sites (K6, 10, 12, 21, 23, 32, 34, 43, and 96) and then incubated with purified 26S proteasome *in vitro*. We found that, in general, mono-ubiquitination supported the degradation of α-synuclein when it was located near the N-terminus of the protein (K6, 10, 12, 21 and 23) but not further towards the center (K32, 34, 43, and 96), demonstrating the location of mono-ubiquitin in likely key for proteasomal degradation of small proteins, including α-synuclein.

Together these chemical experiments support a protective role for ubiquitination in PD, where the modification can both inhibit aggregation and promote turnover of excess α-synuclein. However, because ubiquitinated protein is found in high concentrations in Lewy bodies, further studies are needed to determine whether ubiquitination plays a protective role in early stages of the disease that might become overwhelmed, or if its potential roles are more complicated *in vivo.*

## 6. SUMOylation

The three mammalian isoforms of the Small Ubiquitin-like MOdifier (SUMO1, 2/3) are members of the ubiquitin-like protein family and carry out a range of biological processes including effects on the localization, stability, and protein-protein interactions of their substrates [91,92]. SUMO2 and 3 have 97% sequence identity but only 47% sequence identity with SUMO1; however, all three proteins share the canonical ubiquitin protein-fold. Like ubiquitin, the C-terminus of SUMO is activated by an E1 enzyme, followed by transfer to an E2 and selection of substrates by an E3 enzyme. Additionally, SUMOylation can be removed by several deSUMOylases, rendering the modification dynamic. SUMO is typically added to lysine residues within a consensus motif (ψ-K-x-D/E; ψ = hydrophobic amino acid, x = any amino acid). *In vivo* and in cell culture, α-synuclein is SUMOylated at both one perfect acceptor site (VK^96^KD) and one imperfect lysine (GK^102^NE) [93,94,95]. Additionally, aggregates from PD and dementia with Lewy bodies patients are immunoreactive to SUMO1 [96]. However, SUMO is only found in portions of these aggregates indicating that it may not directly associate with α-synuclein [97,98]. Two separate studies from the same labs found that when the proteasome is inhibited SUMOylation is associated with increased aggregation of α-synuclein [96,99]. In contrast, mutation of the potential SUMOylation sites in α-synuclein (K96 and 102) promoted α-synuclein aggregation in both mammalian cell culture and living yeast [94,100], and a heterogeneous mixture of SUMOylated α-synuclein from an *E. coli* co-expression system was resistant to aggregation *in vitro* [94]. Recently, we used the disulfide-directed strategy to synthesize homogeneously SUMOylated α-synuclein at either K96 or K102 [101]. Notably, we found differences between the two different sites of modification, as well as the isoform of SUMO (*i.e.*, SUMO1 *versus* SUMO2/3). In general, α-synuclein aggregation was more inhibited by modification at K102 when compared to K96, despite them being only 6 residues apart in the primary sequence. Additionally, we found that SUMO1 was a stronger inhibitor than SUMO3 when placed at either modification site. Interestingly, ubiquitination at K96 completely inhibits fiber formation [87], supporting different consequences for individual ubiquitin-like modifiers in α-synuclein aggregation and further mechanistic studies into how these modifications physically prevent aggregation.

## 7. O-GlcNAc Modification

O-GlcNAc modification (O-GlcNAcylation) is the addition of a single monosaccharide to serine and threonine side-chains on intracellular proteins. It is transferred from the high energy sugar-donor UDP-GlcNAc to substrate proteins by O-GlcNAc transferase (OGT) and can be subsequently removed by O-GlcNAcase (OGA) [102]. The global levels of O-GlcNAcylation are linked to changes in cellular metabolism, as OGT modifies different proteins under different levels of UDP-GlcNAc [103]. O-GlcNAcylation has been identified in unbiased proteomics experiments as an α-synuclein modification in mouse neurons at T53 (alanine in humans), T64, and T72 and in human erythrocytes at S87 [35,36]. However, very little is known about the consequences. We took advantage of the fact that the addition of peptides corresponding to the NAC region of α-synuclein will accelerate the aggregation of full-length α-synuclein *in vitro* [104,105]. To test the effect of O-GlcNAcylation, we first synthesized a glycopeptide corresponding to α-synuclein residues 68–77 bearing an O-GlcNAc at T72. Using standard aggregation conditions, we then demonstrated that this glycopeptide did not accelerate the aggregation of α-synuclein, while unmodified peptides from the NAC region did [106], indicating that O-GlcNAcylation at T72 will likely inhibit the aggregation of full-length protein. We are currently in the process of synthesizing O-GlcNAcylated α-synuclein to directly test this hypothesis.

## 8. Nitration

Oxidative stress leads to the production of both reactive oxygen and nitrogen species [107,108]. Some of these reactive nitrogen compounds can result in either direct nitration of tyrosine side-chains to produce 3-nitrotyrosine and the formation of tyrosine-tyrosine covalent crosslinks that generate protein dimers, trimers, and oligomers [109]. Protein aggregates in the brains of patients who suffered from a range of synucleinopathies stain strongly for nitrated α-synuclein [110,111], making the investigation of its effects an important goal. Unfortunately, chemical nitration of recombinant α-synuclein results in a combination of nitration products and tyrosine-tyrosine crosslinks, resulting in the analysis of a heterogenous mixture of proteins. Recently Lashuel and co-workers addressed this limitation using protein semisynthesis [112]. Specifically, they used NCL to prepare α-synuclein with site-specific nitration at either tyrosine 39 or 125. They found that nitration, at either site, resulted in the formation of a mixture of amorphous aggregates and smaller and wider fiber structures, when compared to unmodified α-synuclein. Additionally, they found that nitration has a slight but significant inhibitory-effect on the interaction between α-synuclein and membranes. Finally, they used their semisynthetic proteins to show that pre-nitration of specific tyrosine residues have important effects on the structure and extent of tyrosine-tyrosine crosslinks upon treatment with reactive nitrogen species. Further studies are needed to determine whether nitration is detrimental or beneficial by both direct changes in protein aggregation and consequences on tyrosine-tyrosine crosslinks.

## 9. Conclusions

Significant progress has been made in the identification of α-synuclein posttranslational modifications, and many cellular and *in vivo* experiments have contributed to our understanding of how these modifications may contribute to α-synuclein aggregation and toxicity. These studies typically rely on the expression of α-synuclein mutants that cannot be modified or on overexpression of the enzymes that install the posttranslational modification of interest, which do not test the direct consequences of the modification on α-synuclein’s biophysical properties. Application of peptide and protein chemistry to this problem has enabled the preparation and characterization of several site-specific α-synuclein modifications, including phosphorylation, ubiquitination, and SUMOylation. These studies have identified several posttranslational modifications that inhibit protein aggregation, indicating that the enzymes that remove this modifications might be attractive therapeutics targets in PD. However, challenges to the application of synthetic proteins to understanding the role of α-synuclein modifications in disease remain. For example, traditional protein semisynthesis using NCL and EPL can be technically challenging and typically is only done on relatively small scales (<10 mg of protein product). This should encourage the continued development of selective chemistries that allow for the rapid and facile preparation of modified proteins and their analogs. Additionally, many of the synthetic studies have focused on the consequences of modifications on α-synuclein aggregation and toxicity and have not tested the potential effects on the endogenous roles of α-synuclein (e.g., membrane binding and vesicle trafficking). Future studies should also tackle these important issues to determine whether a modification is likely the alter α-synuclein biology outside of PD. Finally, many of the enzymes that could regulate the posttranslational modifications of α-synuclein likely remain unidentified, which should motivate protein chemists to create synthetic probes that would allow for the identification and validation of these key proteins. Therefore, the continued application of chemistry should remain an active area of investigation, so that it may continue to contribute key insights into the role of α-synuclein modifications in PD.
